# Prostate Cancer Stem Cells: Clinical Aspects and Targeted Therapies

**DOI:** 10.3389/fonc.2022.935715

**Published:** 2022-07-08

**Authors:** Isis Wolf, Christian Gratzke, Philipp Wolf

**Affiliations:** ^1^ Department of Urology, Medical Center-University of Freiburg, Freiburg, Germany; ^2^ Faculty of Medicine, University of Freiburg, Freiburg, Germany

**Keywords:** prostate cancer, prostate cancer stem cells, prostate cancer stem cell hypothesis, prostate cancer stem cell antigens, prostate cancer stem cell therapy

## Abstract

Despite decades of research and successful improvements in diagnosis and therapy, prostate cancer (PC) remains a major challenge. In recent years, it has become clear that PC stem cells (PCSCs) are the driving force in tumorigenesis, relapse, metastasis, and therapeutic resistance of PC. In this minireview, we discuss the impact of PCSCs in the clinical practice. Moreover, new therapeutic approaches to combat PCSCs are presented with the aim to achieve an improved outcome for patients with PC.

## Introduction

Prostate cancer (PC) is the most common cancer and the second leading cause of cancer death in men from industrial countries. More than 1.41 million new cases and more than 375,000 deaths by this tumor are expected worldwide every year (1).

If the tumor is limited to the prostate, a good chance of cure is the surgical resection [radical prostatectomy (RP)] or radiation of the organ. Both treatment options are associated with adverse effects, such as incontinence and sexual dysfunction, which negatively affect the quality of life. The prerequisite for cure is the complete removal of the tumor. If residual tumor cells persist, the tumor may soon relapse and begin to metastasize. Overall, biochemical failure after RP in node-negative patients occurs in approximately 15%–40% of cases within 5 years and is independent of the surgical approach [reviewed in (2)]. The only potentially curative treatment for patients with local recurrence at the earliest sign of biochemical failure is salvage radiation therapy, preferably for PSA levels <0.2 ng/ml (3). In case of metastasized tumors, treatment options include androgen deprivation therapy (ADT) and chemotherapy (4, 5). However, chemo- and castration-resistant PC commonly develop and mainly contribute to therapy failure and mortality (6, 7).

One model that explains heterogeneity, tumor-initiating capability, and therapeutic resistance of tumors is the cancer stem cell (CSC) hypothesis. The CSC hypothesis postulates that cancer cells are hierarchically organized and form different heterogeneous subpopulations within a tumor. CSCs are on top of the hierarchy and represent cancer cells with stem cell-like properties, such as self-renewal, pluripotency, and plasticity, that evolve during the lifetime of a tumor (8, 9). Different factors are discussed that might foster the emergence of CSCs, like de-differentiation through genetic and epigenetic alterations, clonal expansion, and adaptation through epithelial–mesenchymal transition (EMT) as well as transdifferentiation under the influence of the tumor microenvironment or under therapeutic pressure [reviewed in (10)]. CSCs were found to be the driving force in tumor progression, metastasis, and therapeutic resistance, and new strategies are being developed to identify and treat them (11). Our minireview describes clinical aspects of prostate CSCs (PCSCs) and new therapeutic options, aiming to achieve a cure for hitherto incurable stages of the disease.

## Prostate Cancer Stem Cells

PC cells with stem cell characteristics, such as self-renewal, pluripotency, and plasticity, were isolated from patients undergoing RP for the first time in 2005 (12). PCSCs can also be obtained from established PC cell lines, especially of metastatic origin (12–14) and are characterized by sphere formation under non-adherent culture conditions, high clonogenicity, high rate of self-renewal, and the ability to form phenotypically mixed populations of non-clonogenic cells (12, 15). Different antigens, which are involved in various signaling pathways of tumorigenesis, metastasis, and therapeutic resistance of PC, were identified to characterize PCSCs (**Table 1**) (58).

**Table 1:** Antigens associated with PCSCs.

Target antigen	Structure	Function	Role in PC	Ref.
α2β1 integrin	Type I collagen receptor	Cell adhesion, signaling	Self-renewal, proliferation, differentiation, migration, invasion, metastasis	(12, 16, 17)
α_6_ integrin/ D49f	Transmembrane glycoprotein	Cell signaling	Sphere formation, differentiation, tumor progression, invasion	(17–19)
ABCB1	Transmembrane protein	Transporter	Chemoresistance	(20–22)
ABCG2	Transmembrane protein	Transporter	Stem cell maintenance, chemoresistance	(23, 24)
ALDH	Cytosolic enzyme	Aldehyde dehydrogenase	Tumorigenicity, clonogenicity, tumor progression, self-renewal, migration, metastasis, radioresistance ALDH1A1 expression positively correlated with Gleason score and pathologic stage	(25) (26–28) (26)
AR variant 7	Androgen receptor splice variant	Transcription factor	Acquisition of stem cell characteristics, EMT Associated with enhanced progression to mCRPC and shorter survival	(29) (30)
CD117/c-kit	Type III tyrosine kinase receptor	Cell signaling, survival, metabolism, growth, proliferation, apoptosis, migration, differentiation	CSC maintenance, sphere formation, proliferation, migration, invasion, tumor progression, bone metastasis, therapeutic resistance Increased expression during clinical progress	(31) (32)
CD133/Prominin-1	Glycosylated pentaspan transmembrane protein	Precise physiological function unknown	Tumorigenicity, self-renewal, sphere formation, proliferation, differentiation, invasion, chemo/radioresistance	(12, 33–36)
CD166/ activated leukocyte cell adhesion molecule (ALCAM)	Transmembrane glycoprotein	Cell adhesion	Sphere formation, bone metastasis Upregulated in CRPC	(37, 38) (37)
CD44	Transmembrane protein, hyaluronic acid receptor	Cell adhesion, signaling	Self-renewal, proliferation, differentiation, invasion, metastasis	(12, 39)
CXCR4	Chemokine receptor	Chemotaxis, hematopoietic stem cell maintenance	CSC maintenance, clonogenicity, differentiation, migration, metastasis, chemoresistance Overexpressed in metastatic disease	(40–42) (43)
E-cadherin/ ECAD	Transmembrane glycoprotein	Cell adhesion, regulation of epithelial morphogenesis, and differentiation	Sphere formation Expression correlates with recurrence after RP and metastasis	(44) (45, 46)
EpCAM/ CD326	Transmembrane glycoprotein	Cell adhesion, signaling, migration, proliferation, differentiation	CSC maintenance, proliferation, invasion, metastasis, chemo-/radioresistance Overexpressed in local and metastatic disease Overexpressed in chemo-/radioresistant stages	(47, 48) (47) (49, 50)
EZH2 (enhancer of zeste homolog 2)	Cytosolic enzyme	Histone-lysine N-methyltransferase	PCSC maintenance and growth Coactivator for transcription factors in CRPC, including AR Positive EZH2:ECAD status strongly associated with recurrence after RP	(51–53) (53) (53)
TG2 (tissue trans-glutaminase)	Cytosolic enzyme	Protein-glutamine γ-glutamyltransferase	Invasion, chemoresistance, EMT	(54)
Trop2 (trophoblast cell-surface antigen 2)/ tumor-associated calcium signal transducer 2 (TACSTD2)/ epithelial glycoprotein-1 (EGP-1)	Transmembrane protein	Calcium signal transducer	Self-renewal, sphere formation, proliferation, migration, invasion, metastasis Upregulated in invasive stages	(55, 56) (57)

PCSCs might originate from normal prostate stem cells, normal prostate progenitor cells, or differentiated normal prostate cells after genetic and epigenetic alterations or changes in the tumor microenvironment (15). It was found that activation of the proto-oncogene MYC, loss of the tumor suppressor PTEN, or mutations in the repair genes *BRCA2, ATM*, and *CHEK2*, induce genomic instability and drive progression and heterogeneity of PC (10, 59, 60). Polson and colleagues showed a high frequency of the TMPRSS2:ERG gene fusion not only in differentiated PC cells but also in PCSC (61). When the transcription factor ERG comes under the control of the prostate-specific, androgen-regulated TMPRSS2 gene promoter, enhanced ERG expression is found. Since enhanced EGR expression can influence differentiation, self-renewal, and maintenance of SCs, it is discussed that the TMPRSS2: ERG fusion might also play a decisive role in the emergence and maintenance of PCSCs (62).

PCSCs can evolve from basal or luminal epithelial cells after oncogenic transformation (11). Recent findings from a single-cell sequencing study in mice suggest that differentiated luminal cells that survived castration can contribute to prostate regeneration by acquisition of stem cell-like regenerative properties (63). There is also evidence that PCSCs might come from the basal cell layer, because tissue-derived tumor-initiating cells in immunocompromised mice expressed basal markers (such as p63), but did not express the androgen receptor (AR) or markers of luminal differentiation (PSA and PAP) (64). Moreover, there might be a loss of basal cells and expansion of luminal cells during PC tumorigenesis (65). For example, Choi and colleagues demonstrated that inactivation of the tumor suppressor PTEN induced the differentiation of basal cells to transformation-competent luminal cells (66).

PCSCs can differentiate into PC progenitor cells (PCPCs) or differentiated PC cells (DPCCs), which leads to the typical formation of heterogeneous prostate tumors with increasing grading that is determined by the Gleason score. Interestingly, Castellon et al. found highest expression of the stem cell markers CD133, CD44, and ABCG2 in medium-grade Gleason biopsies compared to lower- or higher-grade biopsies or lymph-node and bone metastases (67). This suggests that PCSCs reach a significant number at stages, in which the tumor seems to be confined to the gland and in which surgical treatment or radiation is usually with curative intention. However, many PC patients develop biochemical relapse despite local therapy (68, 69). It is therefore assumed that PCSCs remain in the surgical or radiation area or have already entered blood circulation and colonized lymph nodes or other organs due to their ability to migrate and persist in extra-prostatic tissues (67). Number and signatures of PCSCs in local tumors are therefore discussed as prognostic factors for PC recurrence (70). For example, significantly enhanced expression of the stem cell markers SOX2, OCT4, KLF4, and ABCG2 in recurrent PC tissues in comparison to non-recurrent PC tissues was found after RP (71).

Radiation is a therapeutic option for local disease, recurrence, or advanced PC. However, radioresistance of PC cells is an obstacle for successful radiation therapy. A subpopulation of PC cells with CSC characteristics was found after radiation that was marked by enhanced PI3K/Akt/mTOR signaling (72). PCSCs therefore appear to contribute to the formation of radioresistant tumors.

PC cells metastasize preferentially in lymph nodes, liver, and bones (73). The spine, pelvis, and ribs are the most frequently observed sites of bone metastasis in PC. This distribution is often multifocal, and preferable involvement of the axial skeleton suggests an affinity to the red bone marrow. It seems that the bone marrow is particularly suitable as a metastatic site for PC cells, because it is strongly supplied with a low blood flow rate. In addition, it seems that the bone marrow, which harbors the hematopoietic stem cells, forms a suitable niche for disseminated PCSCs. About 10% of patients already harbor bone metastases at the time of diagnosis and 70%–80% of patients, who relapse after RP, fatally progress to advanced disease with bone metastases. This confirms that there are already subpopulations of PC cells in early-diagnosed, local prostate tumors with stem-cell like properties that are able to disseminate and colonize distant organs. Metastatic PC cells are marked by a high expression of integrins that promote their adherence to a broad spectrum of proteins of the bone extracellular matrix, and release factors (FGFs, IGFs, VEGF, or Wnt pathway-related factors, originally involved in bone formation and maintenance) for persistence [reviewed in (74)]. Castellon and colleagues found only low expression of PCSC markers in metastases from lymph nodes and bone, but explained this phenomenon with prevalence of PCPCs and DPCCs (67).

PCSCs are marked by low or lack of androgen receptor (AR) expression (75) and, as a result, by a missing or reduced PSA release. Therefore, they might escape a PSA screening and androgen receptor expression and measurable PSA values might only be detected when the metastatic PCSCs have already differentiated and expanded. This could be an explanation for the often observed discrepancy between detection of biochemical recurrence (defined by a rising PSA profile) and already existing progressive disease (76).

First-line treatment of advanced PC is ADT. In general, tumors initially respond well to ADT; however, the therapeutic effect of ADT only lasts for a limited period of 12–33 months. At that point, ADT-resistant PC cells emerge and form castration-resistant tumor lesions (77). There is growing evidence that PCSCs contribute to this phenomenon. Since PCSCs were found to be AR-negative and have the ability to grow androgen independently, they might have a survival benefit when treated with ADT (78). Indeed, whereas AR^+^/PSA^+^ tumor cells form the main cell population in untreated androgen-sensitive tumors, enrichment of AR^-/lo^ and PSA^-/lo^ cells was found in untreated higher grades of the disease and in CRPC (79). Moreover, genes associated with CSCs, like *OCT4, SOX2, NANOG, BMI1, BMP2, CD44, SOX9*, and *ALDH1*, were found to be upregulated in enzalutamide-resistant cells (80). There is evidence that truncated AR variants, which lack the ligand binding domain, but retained transcriptional activity, play a decisive role. In particular, the variant AR-V7 might be involved in EMT and promotes the emergence of PCSCs (81).

Re-programming of PC cells to stem-like cells during ADT was demonstrated in a recent study. After androgen depletion over 90 days, a re-differentiation to a stem-like phenotype was observed in LNCaP cells, which was marked by growth as floating spheres and enhanced expression of CD133, ALDH1A1, and the multidrug resistance protein transporter ABCB1A. Interestingly, ABCB1A expression in the re-differentiated stem-like cells was associated with enhanced resistance against docetaxel and 2-hydroxyflutamide (20). This provides a rationale that chemoresistance may already be induced in prostate tumors during ADT and reinforces the medical guidelines recommending chemotherapy in hormone-naïve PC (82).

An example for transdifferentiation is the emergence of neuroendocrine PC cells (NEPCCs) in about 25% of patients after treatment with ADT (83). NEPCCs are hormone-refractory and secrete peptide hormones and growth factors for paracrine stimulation of surrounding cells in the microenvironment (83). It was found that loss of PTEN concurrently with inactivation of the tumor suppressors TP53 and Rb1 caused plasticity of PC cells with enhanced metastatic potential and conversion from adenocarcinoma to neuroendocrine PC [reviewed in (84)].

PCSCs also contribute to chemoresistance of PC. Docetaxel (DTX)-resistant cells showed enhanced expression of CD44 and CD133, leading to enhanced migration and invasion (85–87). Moreover, enhanced activity of Notch signaling was found, which promoted DTX resistance by upregulation of ABCC1 (88).

## Targeting PCSCs

Current treatments against PC, like ADT, chemotherapy, and radiotherapy, aim to remove bulk tumors, but do not seem to affect resistant PCSCs effectively. Therefore, research is increasingly being conducted into new therapeutic approaches against PCSCs. Such approaches comprise the targeting of PCSC-associated pathways, the targeting of the PCSC microenvironment, and immunotherapies.

### Targeting PCSC-Associated Signaling Pathways

The Hedgehog (Hh), Wnt, Notch, and NF-kB pathways, which regulate proliferation, survival, metastasis, apoptosis, recurrence, and therapeutic resistance, were identified to be associated with PCSCs (11, 89–91). Different strategies have therefore been developed to target these pathways by inhibitors or RNA silencing (90, 92–94). Specific inhibitors against the Hh pathway (Sonidegib, GANT-61, and GDC-0449), the Wnt pathway (3289-8625, LGK974, Foxy-5, and OMP-54F28), the Notch pathway (RO4929097), and the NFkB pathway [bortezomib, PS1145, BMS345541, Aspyrin, 17-(allylamino)-17-demethoxygeldanamycin, and BKM120] are tested in preclinical and clinical trials with the intention of attacking PCSCs for an improved therapeutic outcome (90).

The PI3K/AKT/mTOR pathway is associated with PC progression and ADT resistance (95). Suppression of this pathway is therefore discussed to restore sensitivity against ADT, chemotherapy, and radiation (96, 97). In a recent study, an enhanced sensitivity of LNCaP cells against paclitaxel was determined after siRNA knockdown of the stem cell marker CD133. Mechanistically, an induction of the tumor suppressor PTEN accompanied by a decrease of AKT and c-myc oncogenes was found (33). Chang and colleagues were able to restore radiosensitivity and to induce apoptosis in radioresistant PCSCs by the use of the dual PI3K/mTOR inhibitor BEZ235 (72). Marhold and colleagues found elevated HIF1α levels and an enhanced HIF target gene expression in PCSCs under hypoxic conditions. This was accompanied by drug resistance to selective mTOR inhibitors. The authors therefore proposed a deregulation of the PI3K/AKT/mTOR pathway through HIF1α for quiescence and maintenance of PCSCs by attenuating CSC metabolism and growth *via* mTOR signaling and promoting survival by AKT signaling (98). Since hypoxia often prevails in the tumor microenvironment, targeting the HIF1α pathway might damage PCSCs while sparing normal stem cells.

ABC transporters were found to contribute to drug resistance of PSCs (99) and PCSCs (67). Liu and colleagues examined that the intracellular domain of NOTCH1, called ICN1, directly binds to the promoter region of ABCC1 and that inhibition of NOTCH1 with shRNA decreased ABCC1 expression and restored chemosensitivity of PCSCs (88). ABCG2 was found to play a decisive role in ADT-resistant PSCs by efflux of intracellular androgens. When ABCG2 was blocked by the inhibitor Ko143, an increasing nuclear AR level was observed followed by enhanced AR regulated gene expression and increased differentiation with ADT-sensitive luminal phenotype (23). Future experiments have to prove whether targeted differentiation is a new strategy to sensitize PCSCs to conventional therapies.

### Targeting the PCSC Microenvironment

Multiple signaling pathways exist between epithelial cells, stromal cells, and the extracellular matrix of the prostate tumor microenvironment to support tumor progression from the primary site to regional lymph nodes and distant metastases. For example, the CSC niche was found to induce Hh, Wnt, NF-κB, Notch, or PI3K/AKT/mTOR signaling in CSCs (100). Therefore, targeting of these pathways aims to disrupt the interaction between the microenvironment and the tumor cells in order to stop tumor spread [reviewed in (100, 101)].

Since PCSCs are also dependent on a microenvironment, called the PCSC niche, for the maintenance of their stemness properties, research is ongoing to investigate whether targeting of the tumor microenvironment might also lead to damage of PCSCs (11). The monoclonal antibody bevacizumab can be used to target the vascular endothelial growth factor (VEGF) and to reduce the tumor neovasculature for disruption of CSC niches. Bevacizumab-resistant PCSCs were found to have Rac1-mediated ERK activation, and Rac1 inhibition or P-Rex1 downregulation increased their sensitivity to bevacizumab (102). The CXCL12/CXCR4 chemokine pathway was also found to be activated in CD44/CD133-positive PCSCs and to affect cell adhesion, clonal growth, and tumorigenicity. The use of the CXCR4 antagonist AMD3100 inhibited sphere formation and restored the chemosensitivity of PCSCs (103). Since CD44 associates with the extracellular matrix hyaluronic acid (HA) (104), HA-coated liposomes containing cabazitaxel were generated for the inhibition of migration and the triggering of apoptosis in CD44-positive PC cells (105).

### Immunotherapy of PCSCs

PCSCs show enhanced expression of cell surface markers that can serve as target antigens for new immunotherapeutic approaches (**Table 1**). Recently, chimeric antigen receptor (CAR)-modified T-cell therapy targeting CSC-associated tumor antigens emerged as a new therapeutic approach for the treatment of CSCs (106). Zhu et al. demonstrated that anti-C133 CAR-T therapy leads to toxicity of patient-derived glioblastoma CSCs *in vitro* and in an orthotropic tumor model *in vivo* (107). Another study by Deng et al. showed that CAR T cells targeting the CSC marker EpCAM reduced PC progression in preclinical models (49). Currently, there is only one completed phase I/II clinical study of CD133-directed CAR T cells for the treatment of relapsed and/or chemotherapy refractory advanced hepatocellular carcinoma (NCT04427449) (108). However, several clinical trials using CAR-T cells targeting CSC surface markers are in the recruiting stage, representing a promising therapeutic option for the treatment of PCSCs in the future (106).

A further immunotherapeutic approach includes the use of dendritic cells preloaded with the PCSC-associated antigens CD44 and EpCAM for the activation of cytokine-induced killer T cells. This led to high cytotoxicity against PCSCs *in vitro* and antitumor activity *in vivo* in PCSC-derived xenograft models (109). Ma and colleagues generated aptamer-based liposomes loaded with curcumin to target CD133-positive PC cells and found antitumor activity in a PC mouse xenograft model (110). Interestingly, CD133 is expressed on both CSCs and differentiated tumor cells, but seems to be differentially folded and glycosylated, and therefore presents different target epitopes (111). Since different antibodies recognize different glycosylated CD133 epitopes (112), evaluation of glycosylation patterns of markers in PCSCs and differentiated PC cells could lead to the development of antibodies specifically directed against PCSCs in the future. Other strategies comprise the targeting of multiple antigens to enhance PCSC specificity or the targeting of potentially relevant splice variants.

## Conclusions

PCSCs were identified as the driving force in PC. There is emerging knowledge about the role of PCSCs, and new therapeutic approaches aim to achieve an improved therapeutic outcome. Selective and effective targeting of PCSCs, however, remains challenging, since cellular plasticity and intra- as well as inter-tumoral heterogeneity drive tumor progression and therapeutic resistance against conventional therapies (113, 114). From a clinical perspective, the understanding of the interactions between PCSCs, differentiated PC cells, and the TME is of utmost importance, but these interactions are very difficult to reproduce *in vitro*. Furthermore, some CSC markers (e.g., CD133 and ALDH) are expressed not only on malignant cells but also on healthy stem cells causing on-target/off-tumor toxicity (115, 116). Therefore, treatment side effects can be hindrances for a successful therapy of PCSCs. In the future, the characterization of PCSCs using (single-cell) genomics and proteomics could lead to improved prognosis and more individualized therapy for patients with PC to probably achieve complete cure of advanced hitherto incurable stages of the disease.

## Author Contributions

PW drafted the manuscript and designed the figure. IW provided the data for the table. IW, CG, and PW wrote, discussed and reviewed the final manuscript. All authors contributed to the article and approved the submitted version.

## Funding

This work was supported by a grant from the German Research foundation (Grant No. WO2178/3-1 to PW). We acknowledge support by the Open Access Publication Fund of the University of Freiburg.

## Conflict of Interest

The authors declare that the research was conducted in the absence of any commercial or financial relationships that could be construed as a potential conflict of interest.

## Publisher’s Note

All claims expressed in this article are solely those of the authors and do not necessarily represent those of their affiliated organizations, or those of the publisher, the editors and the reviewers. Any product that may be evaluated in this article, or claim that may be made by its manufacturer, is not guaranteed or endorsed by the publisher.

## Abbreviations

ADT, androgen deprivation therapy; AR, androgen receptor; ABCG2, ATP-binding cassette sub-family G member 2 transporter; CSC, cancer stem cell; CRPC, castration-resistant prostate cancer; DPCC, differentiated prostate cancer cells; EMT, epithelial–mesenchymal transition; NEPCC, neuroendocrine prostate cancer cell; PAP, prostatic acid phosphatase; PCPC, prostate cancer progenitor cells; PCSC, prostate cancer stem cell; RP, radical prostatectomy; PSA, prostate-specific antigen.
